# Electron beam characteristics at extended source-to-surface distances for irregular cut-outs

**DOI:** 10.4103/0971-6203.71763

**Published:** 2010

**Authors:** T. Arunkumar, Sanjay S. Supe, M. Ravikumar, S. Sathiyan, M. Ganesh

**Affiliations:** Department of Radiation Physics, Kidwai Memorial Institute of Oncology, Hosur Road, Bangalore, India

**Keywords:** Cerrobend cutout, electron beam, flatness, isodose, uniformity index

## Abstract

Electron beam therapy is widely used in the management of cancers. The rapid dose fall-off and the short range of an electron beam enable the treatment of lesions close to the surface, while sparing the underlying tissues. In an extended source-to-surface (SSD) treatment with irregular field sizes defined by cerrobend cutouts, underdosage of the lateral tissue may occur due to reduced beam flatness and uniformity. To study the changes in the beam characteristics, the depth dose, beam profile, and isodose distributions were measured at different SSDs for regular 10 × 10 cm^2^ and 15 × 15 cm^2^ cone, and for irregular cutouts of field size 6.5 × 9 cm^2^ and 11.5 × 15 cm^2^ for beam energies ranging from 6 to 20 MeV. The PDD, beam flatness, symmetry and uniformity index were compared. For lower energy (6 MeV), there was no change in the depth of maximum dose (R100) as SSD increased, but for higher energy (20 MeV), the R_100_ depth increased from 2 cm to 3 cm as SSD increased. This shows that as SSD increases there is an increase in the depth of the maximum dose for higher energy beams. There is a +7 mm shift in the R_100_ depth when compared with regular and irregular field sizes. The symmetry was found to be within limits for all the field sizes as the treatment distance extended as per International Electro technical Commision (IEC) protocol. There was a loss of beam flatness for irregular fields and it was more pronounced for lower energies as compared with higher energies, so that the clinically useful isodose level (80% and 90%) width decreases with increase in SSD. This suggests that target coverage at extended SSD with irregular cut-outs may be inadequate unless relatively large fields are used.

## Introduction

Electron beam therapy is widely used in the management of cancers. The rapid dose fall-off and the short range of an electron beam enable the treatment of lesions close to the surface, while sparing the underlying tissues. The irregular shapes of individual tumors, however, require the need for custom-made cut-outs so as to conform the shape of the radiation field to that of the tumor, while sparing radiation to surrounding tissues. Electron beam treatments are occasionally performed at extended SSD of 101–120 cm as the body anatomy may obstruct the positioning of the applicator[1–3]. As there would be electron contamination whenever electron cut-outs are used, the beam parameters such as percentage depth dose (PDD), beam profiles, and isodose curves must be measured every time. Extended treatment distances present the problem of changes in the beam characteristics. Most treatment planning systems are unable to provide dose distribution accurately for clinical use. The goal of this study is to investigate the correlation of electron beam characteristics, such as percent depth dose curves, beam profile, and isodose distribution between regular square cones and cut-outs of irregular field sizes inserted into the cones at nominal and extended SSDs for various electron beams in the range from 6 to 20 MeV that are available with the machine.

## Materials and Methods

The machine used for measurements in this study was a Clinac 2100 – DHX linear accelerator (Varian Medical Systems, Palo Alto, CA). It provides dual photon energies of 6 MV and 18 MV, as well as electron energies of 6, 9, 12, 16, and 20 MeV. The machine is isocentrically mounted with an SAD (source-axis distance) of 100 cm. The cut-outs used in this work were made of cerrobend, which is a low-temperature melting alloy containing bismuth, lead, tin, and cadmium (50.0%, 26.7%, 13.3%, and 10.0% by weight, respectively), placed at the end of the applicator. The required shielding thickness of the cut-outs should he approximately equal to the maximum range of the highest electron energy beam available in cerrobend. Therefore, each has a thickness of 1.6 cm, which will reduce the transmitted dose to <10%. The experiment was carried out for 10 × 10 cm^2^ and 15 × 15 cm^2^ cones. The cerobend cut-out[4] defining irregular field, which was designed for the patient treatment, is inserted in the cone. For 10 × 10 cm^2^ cone, the cut-out dimension used was 6.5 × 9.0 cm^2^ and, for 15 × 15 cm^2^ cone, the cut-out dimension was 11.5 × 15 cm^2^. The percentage depth dose and beam profile along the central axis were measured using radiation field analyzer (RFA 300, Scanditronix Wellhofer, Germany) with a p-type silicon diode in water phantom at nominal (SSD = 100 cm) and at extended SSD (102–120 cm). Both in-line and cross-line beam profiles were measured. The beam profiles were measured at six depths R100, R90, R80, R50,practical range (Rp), and therapeutic range (Rt = depth of 85% dose). The relative surface dose has been taken from the PDD curves at 0.05 cm down from the water surface, in order to avoid possible errors at the air–water interface. The isodose curves along the beam axis were generated by the OmniPro-Accept software using the PDD curve and the beam profile according to the Bently’s beam model. Beams eye view (BEV) isodose curves perpendicular to the beam central axis were measured using I’matriXX device (Scanditronix Wellhofer, Germany). The effective point of measurement is at 0.36 cm from the surface of the device. The plane selected here for the isodose measurement was at a depth of half of the therapeutic range, i.e., ½ R _85_, and the dose in this plane was normalized to 100% at the center. The beam’s eye view isodose was measured to calculate the uniformity index (UI _90/50_).[5] The uniformity index is defined as the ratio of area inside 90% and 50% isodose line. All the measurements were carried out for nominal and extended SSDs.

## Results and Discussion

### Percentage depth dose

Figure 1a–d shows a series of PDD curves obtained using the 6.5 × 9.0 cm^2^ and 11.5 × 15 cm^2^ cut-outs for electron beam energies of 6 and 20 MeV. The shapes of the PDD curves are characteristic of clinical electron beams. Each PDD displays a high surface dose, a buildup region, a broad dose maximum, a sharp dose fall-off, and a bremsstrahlung tail. These results are illustrated in the Tables 1a and Table 1b. Based on these datasets, the following conclusions can be made: The depth of dose maximum R _100_ for 6 MeV which is 1.4 cm remains constant for regular (10 × 10 cm^2^ and 15 × 15cm^2^) as well as for irregular field sizes (6.5 × 9 cm^2^ and 11.5 × 15 cm^2^) as the treatment distance increases. For 20 MeV the R _100_ depth increased from 2 cm to 3.5 cm as the distance increases. There was a +7 mm shift in the R _100_ depth when compared with regular and irregular field sizes as the treatment distance increased. The change in depth dose curve for higher energies was because of large angular scattering of the electron beams. The relative surface dose increases with increase in energy and decreases with increase in SSD, irrespective of field size, by 76.4%–73.9% for 6 MeV and by 92%–88% for 20 MeV. It was noticed that the change in the depth dose curve was minimum and the bremsstrahlung dose component D _x_ = 0.3 for 6 MeV and remains unaltered as the treatment distance increases. But for 20 MeV, the D _x_ increased from 4.6% to 5.4% as the treatment distance increased. These values are in agreement with TG-25.[6] The increase in D_x_ at larger SSD for 20 MeV electron beam may be because of the lesser absorption of low-energy scattered electrons produced from the cerrobend cutout that contributes to the point of measurement.[7]

**Table 1a:** Characteristics of 6-MeV electron beam for regular and irregular field size

*SSD*	*10 × 10 cm^2^*			*6.5 × 9.0 cm^2^*			*15 × 15 cm^2^*			*11.5 × 15 cm^2^*		
	*R_100_ cm*	*D_s_ %*	*D_x_ %*	*R_100_ cm*	*D_s_ %*	*D_x_ %*	*R_100_ cm*	*D_s_ %*	*D_x_ %*	*R_100_ cm*	*D_s_ %*	*D_x_ %*
100	1.3	76.2	0.3	1.4	76.0	0.3	1.4	76.4	0.3	1.4	76.4	0.3
102	1.4	75.2	0.3	1.4	75.3	0.3	1.4	75.7	0.3	1.4	76.3	0.3
104	1.4	75.1	0.3	1.4	75.0	0.3	1.4	75.2	0.3	1.4	75.3	0.3
106	1.4	75.0	0.3	1.4	75.0	0.3	1.4	75.2	0.3	1.4	75.3	0.3
108	1.4	74.5	0.3	1.4	74.5	0.3	1.4	74.7	0.3	1.4	75.2	0.3
110	1.4	74.2	0.3	1.4	74.4	0.3	1.5	74.0	0.3	1.4	75.0	0.3
115	1.4	74.1	0.3	1.4	74.6	0.4	1.4	74.2	0.3	1.4	74.6	0.4
120	1.4	73.9	0.3	1.4	75.0	0.3	1.4	74.5	0.3	1.4	74.5	0.3

**Table 1b:** Characteristics of 20-MeV electron beam for regular and irregular field size

*SSD*	*10 × 10 cm^2^*			*6.5 × 9.0 cm^2^*			*15 × 15 cm^2^*			*11.5 × 15 cm^2^*		
**	*R_100_ cm*	*D_s_ %*	*D_x_ %*	*R_100_ cm*	*D_s_ %*	*D_x_ %*	*R_100_ cm*	*D_s_ %*	*D_x_ %*	*R_100_ cm*	*D_s_ %*	*D_x_ %*
100	2.0	92.8	4.6	1.9	92.6	4.6	2.2	92.0	4.9	2.0	92.1	4.9
102	2.4	91.8	4.7	2.1	91.5	4.6	2.2	91.3	5.0	2.2	91.4	5.1
104	2.5	91.2	4.8	2.1	90.9	4.7	2.6	90.8	5.0	2.4	90.6	5.0
106	2.7	90.7	4.8	2.2	90.6	4.7	3.0	90.5	5.1	2.7	90.4	5.0
108	2.7	89.9	4.9	2.2	89.8	4.8	2.7	90.2	5.1	3.0	89.2	5.1
110	2.9	89.2	4.9	2.7	89.1	4.8	3.0	89.8	5.1	2.7	89.6	5.1
115	3.2	89.0	5.0	3.0	88.1	4.9	3.0	88.6	5.2	2.9	88.9	5.1
120	2.9	88.0	5.1	3.0	88.3	5.0	3.5	88.1	5.4	2.8	88.4	5.2

**Figure 1a:**
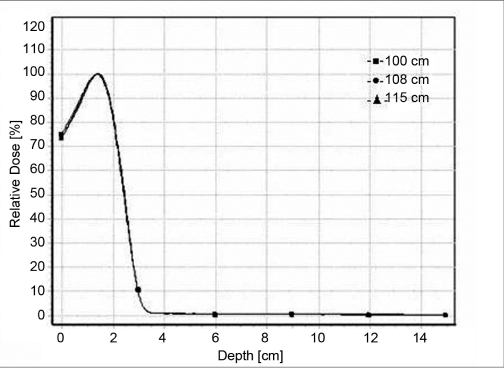
Depth dose curves for 6-MeV electron beam at 100 cm FSD, 108 cm FSD and 115 cm FSD for 6.5 × 9.0 cm^2^ field size

**Figure 1b:**
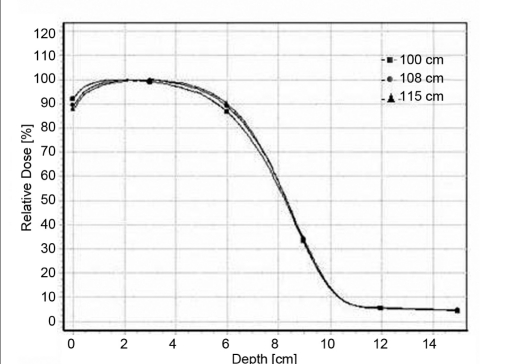
Depth dose curves for 20-MeV electron beam at 100 cm FSD, 108 cm FSD and 115 cm FSD for 6.5 × 9.0 cm^2^ field size

**Figure 1c:**
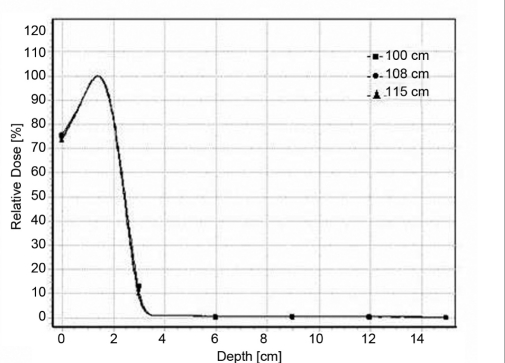
Depth dose curves for 6-MeV electron beam at 100 cm FSD, 108 cm FSD and 115 cm FSD for 11.5 × 15 cm^2^ field sized

**Figure 1d:**
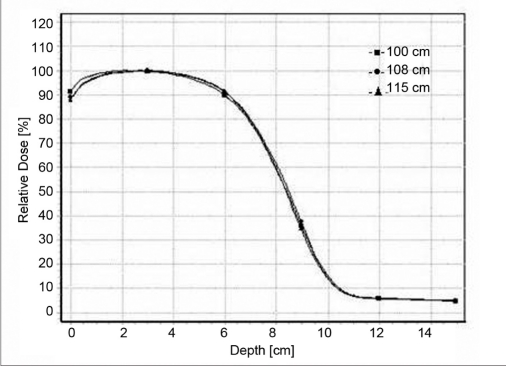
Depth dose curves for 20-MeV electron beam at 100 cm FSD, 108 cm FSD and 115 cm FSD for 11.5 × 15 cm^2^ field size

### Beam profiles

Profiles for 6.5 × 9.0 cm^2^ and 11.5 × 15 cm^2^ for 6 MeV and 20 MeV are shown in the Figures 2–d. Both inline and cross-line profiles were measured. For regular field size there was no variation between the inline and cross-line profiles, but for irregular field sizes there was significant variation.

**Figure 2a:**
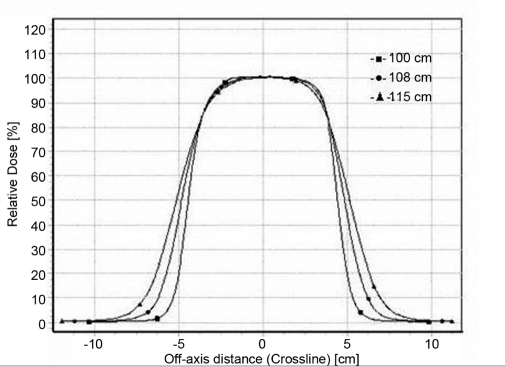
Cross-line profiles for 6-MeV electron beam at 100 cm FSD, 108 cm FSD and 115 cm FSD for 6.5 × 9 cm^2^ field size

**Figure 2b:**
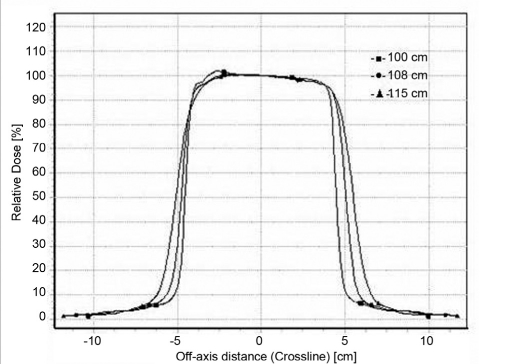
Cross-line profiles for 20-MeV electron beam at 100 cm FSD, 108 cm FSD and 115 cm FSD for 6.5 × 9 cm^2^ field size

**Figure 2c:**
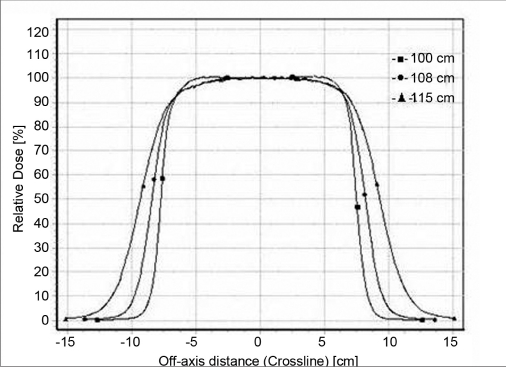
Cross-line profiles for 6 MeV electron beam at 100 cm FSD, 108 cm FSD and 115 cm FSD for 11.5 × 15 cm^2^ field size

**Figure 2d:**
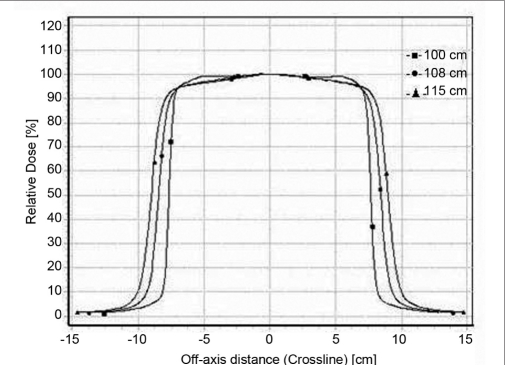
Cross line profiles for 20-MeV electron beam at 100 cm FSD, 108 cm FSD and 115 cm FSD for 11.5 × 15 cm^2^ field size

### Symmetry and flatness

Table 2a–d shows the symmetry, flatness, and penumbra values for 6 MeV and 20 MeV for regular field sizes 10 × 10 cm^2^, 15 × 15 cm^2^, and for irregular field sizes 6.5 × 9.0 cm^2^ and 11.5 × 15 cm^2^. The flatness and symmetry were evaluated based on International Electrotechnical Commission (IEC)[8] specification. According to the protocol, the beam flatness requires that the maximum distance between the 90% dose and the edges of the geometrical field shall be <10 mm along the principal axis. The symmetry of the beam is measured by the difference in dose at two points placed symmetrically from the central axis and should be ≤3%. The symmetry was found to be within limits for all the field sizes as the treatment distance extended as per IEC protocol. At extended SSD, particularly at dmax loss of electron beam flatness characterized by a round shape profile was observed. For 6-MeV energy the flatness varies from 0.76 cm to 1.81 cm for 10 × 10 cm^2^ field size, 2.56 cm to 3.91 cm for 6.5 × 9.0 cm^2^ (in-line profile) field size, and 1.74 cm to 2.89 cm^2^ (cross-line profile) as the distance increased from 100 to 120 cm. Similarly, for 20 MeV energy, the flatness varies from 0.26 cm to 0.76 cm for 10 × 10 cm^2^ field size, 2.08 cm to 2.93 cm for 6.5 × 9.0 cm^2^ (in-line profile) field size, and 0.88 cm to 1.76 cm^2^ (cross-line profile) as the distance increased from 100 to 120 cm. For 6-MeV energy, the flatness varies from 0.72 cm to 1.91 cm for 15 × 15 cm^2^ field size, 2.54 cm to 4.14 cm for 11.5 × 15 cm^2^ (in-line profile) field size, and 0.86 cm to 2.03 cm^2^ (cross-line profile) as the distance increased from 100 to 120 cm. For 20-MeV energy, the flatness varies from 0.20 cm to 0.72 cm for 15 × 15 cm^2^ field size, 2.15 cm to 3.08 cm for 11.5 × 15 cm^2^ (in-line profile) field size, and 0.26 cm to 0.80 cm^2^ (cross-line profile) as the distance increased from 100 to 120 cm. The reduction in flatness as the treatment distance increased for regular and irregular field sizes was due to the large angular scattering of electrons in the air medium for lower energy (6 MeV). However, for higher energy (20 MeV) the flatness was better because of the forward scattering of the electrons. Similarly, the flatness was reduced for irregular fields because of the electron contamination from the cerrobend cut-out.

**Table 2a:** Symmetry, flatness and penumbra for 6-MeV electron beam for 10 × 10 cm^2^ and 6.5 × 9.0 cm^2^ field sizes

*SSD*	*Cross-line profile*			*In-line profile*			*Cross-line profile*		
**	*10 × 10 cm^2^*			*6.5 × 9.0 cm^2^*			*6.5 × 9.0 cm^2^*		
**	*Symmetry %*	*Flatness cm*	*Penumbra cm*	*Symmetry %*	*Flatness cm*	*Penumbra cm*	*Symmetry %*	*Flatness cm*	*Penumbra cm*
100	101.2	0.76	1.04, 1.11	100.3	2.56	1.02, 1.11	100.9	1.74	1.24, 1.08
102	100.8	0.80	1.17, 1.20	100.3	2.70	1.18, 1.27	100.5	1.70	1.34, 1.25
104	100.4	0.88	1.29, 1.34	100.4	2.84	1.33, 1.41	101.1	1.80	1.49, 1.41
106	100.5	0.99	1.45, 1.50	100.3	2.98	1.49, 1.56	101.5	1.99	1.71, 1.57
108	100.7	1.10	1.62, 1.68	100.2	3.12	1.66, 1.72	101.9	2.19	1.93, 1.75
110	100.4	1.21	1.78, 1.82	100.5	3.23	1.80, 1.85	100.7	2.18	2.02, 1.91
115	100.3	1.52	2.25, 2.27	100.4	3.61	2.24, 2.31	101.9	2.58	2.51, 2.31
120	100.5	1.81	2.70, 2.69	100.4	3.91	2.66, 2.74	101.7	2.89	2.95, 2.75

**Table 2b:** Symmetry, flatness and penumbra for 20-MeV electron beam for 10 × 10 cm^2^ and 6.5 × 9.0 cm^2^ field sizes

*SSD*	*Cross-line profile*			*In-line profile*			*Cross-line profile*		
**	*10 × 10 cm^2^*			*6.5 × 9.0 cm^2^*			*6.5 × 9.0 cm^2^*		
**	*Symmetry %*	*Flatness cm*	*Penumbra cm*	*Symmetry %*	*Flatness cm*	*Penumbra cm*	*Symmetry %*	*Flatness cm*	*Penumbra cm*
100	101.7	0.26	0.52, 0.56	100.4	2.08	0.50, 0.56	103.0	0.88	0.55, 0.54
102	100.4	0.31	0.64, 0.66	100.8	2.17	0.58, 0.64	102.7	0.94	0.62, 0.60
104	100.5	0.37	0.72, 0.77	101.4	2.25	0.64, 0.70	102.5	1.01	0.69, 0.68
106	100.4	0.43	0.81, 0.87	101.6	2.33	0.71, 0.76	102.4	1.09	0.78, 0.76
108	100.4	0.46	0.84, 0.90	101.5	2.40	0.76, 0.81	102.4	1.12	0.81, 0.81
110	100.7	0.53	0.93, 1.00	101.4	2.55	0.97, 0.98	101.2	1.39	1.10, 1.00
115	100.4	0.68	1.20, 1.23	101.6	2.75	1.20, 1.18	101.9	1.59	1.29, 1.22
120	101.0	0.76	1.27, 1.26	101.5	2.93	1.34, 1.31	101.4	1.76	1.43, 1.36

**Table 2c:** Symmetry, flatness, and penumbra for 6-MeV electron beam for 15 × 15 cm^2^ and 11.5 × 15.0 cm^2^ field sizes

*SSD*	*Cross-line profile*			*In-line profile*			*Cross-line profile*		
**	*15 × 15 cm^2^*			*11.5 × 15.0 cm^2^*			*11.5 × 15.0 cm^2^*		
**	*Symmetry %*	*Flatness cm*	*Penumbra cm*	*Symmetry %*	*Flatness cm*	*Penumbra cm*	*Symmetry %*	*Flatness cm*	*Penumbra cm*
100	100.6	0.72	1.06, 1.06	102.3	2.54	1.03, 1.05	101.7	0.86	1.02, 1.03
102	100.6	0.80	1.16, 1.19	101.7	2.70	1.18, 1.21	101.4	0.90	1.17, 1.18
104	100.3	0.88	1.34, 1.36	101.7	2.83	1.34, 1.36	101.5	1.06	1.34, 1.36
106	100.4	0.99	1.46, 1.50	101.5	3.00	1.53, 1.54	101.6	1.15	1.49, 1.51
108	101.1	1.14	1.61, 1.70	101.7	3.14	1.73, 1.74	101.5	1.30	1.72, 1.71
110	100.9	1.27	1.82, 1.88	101.6	3.28	1.88, 1.88	101.5	1.47	1.91, 1.89
115	100.8	1.56	2.28, 2.33	101.4	3.68	2.34, 2.39	101.6	1.58	2.36, 2.33
120	100.9	1.91	2.77, 2.86	101.5	4.14	2.82, 2.87	101.7	2.03	2.81, 2.81

**Table 2d:** Symmetry, flatness, and penumbra for 20-MeV electron beam for 15 × 15 cm^2^ and 11.5 × 15.0 cm^2^ field sizes

*SSD*	*Cross-line profile*			*In-line profile*			*Cross-line profile*		
**	*15 × 15 cm^2^*			*11.5 × 15.0 cm^2^*			*11.5 × 15.0 cm^2^*		
**	*Symmetry %*	*Flatness cm*	*Penumbra cm*	*Symmetry %*	*Flatness cm*	*Penumbra cm*	*Symmetry %*	*Flatness cm*	*Penumbra cm*
100	100.6	0.20	0.56, 0.57	102.4	2.15	0.51, 0.54	101.1	0.26	0.50, 0.51
102	100.7	0.25	0.58, 0.60	102.5	2.24	0.61, 0.66	100.7	0.31	0.57, 0.61
104	100.5	0.34	0.73, 0.77	102.2	2.36	0.74, 0.77	100.7	0.39	0.68, 0.73
106	100.4	0.45	0.91, 0.95	102.1	2.51	0.90, 0.90	100.4	0.50	0.83, 0.86
108	100.9	0.44	0.88, 0.92	102.1	2.69	1.04, 1.06	100.4	0.57	0.99, 1.01
110	101.0	0.53	1.00, 1.04	101.2	2.69	1.00, 1.02	100.5	0.55	0.97, 0.97
115	101.2	0.59	1.15, 1.13	102.1	2.93	1.19, 1.22	100.8	0.69	1.19, 1.15
120	101.0	0.72	1.42, 1.41	102.2	3.08	1.30, 1.35	101.0	0.80	1.31, 1.28

### Penumbra

Penumbra is the average distance separating the 80% and 20% isodose lines. The electron field penumbra (20%–80% intensity) increased at lower energies and decreased for higher energies. The penumbra increased with increase in the SSD. At high energies, the beam is more forward scattered, with less lateral scattering, giving rise to a narrow penumbra. This is expected because high-energy electrons are subject to less scattering.

### Isodose

Figures 3a–d show the isodose distribution along the central axis for a 6-MeV beam for irregular field sizes at 100 cm SSD and 120 cm SSD. Isodose bulging was observed with increase in treatment distance. It can be seen that there is a reduction in the field flatness. Figure 4–d show the isodose distribution perpendicular to the beam central plane for a 6-MeV beam for 6.5 × 9.0 cm^2^ and 11.5 × 15 cm^2^ field sizes at 100 cm and 120 cm SSD. The figure shows the irregular shape of thecut-out used and the divergence of the isodose lines as the SSD increased.

**Figure 3a:**
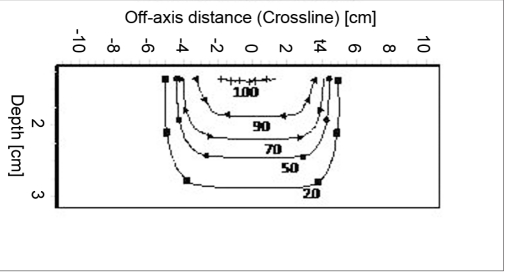
Isodose curves for 6-MeV electron at 100 cm SSD for 6.5 × 9 cm^2^ field size

**Figure 3b:**
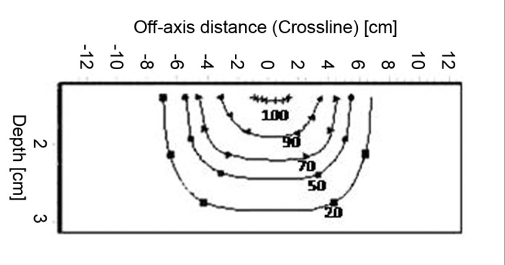
Isodose curves for 6-MeV electron beam at 120 cm SSD for 6.5 × 9 cm^2^ field size

**Figure 3c:**
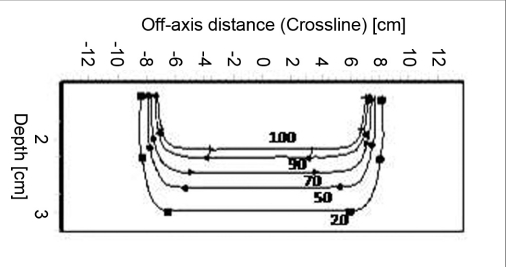
Isodose curves for 6-MeV electron beam at 100 cm SSD for 11.5 × 15 cm^2^ field size

**Figure 3d:**
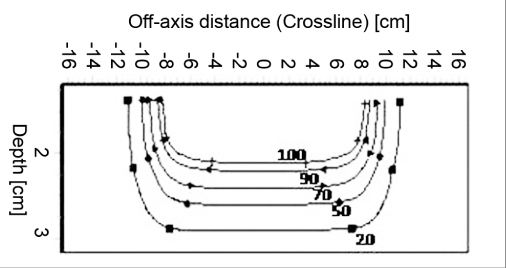
Isodose curves for 6-MeV electron beam at 120 cm SSD for 11.5 × 15 cm^2^ field size

**Figure 4a:**
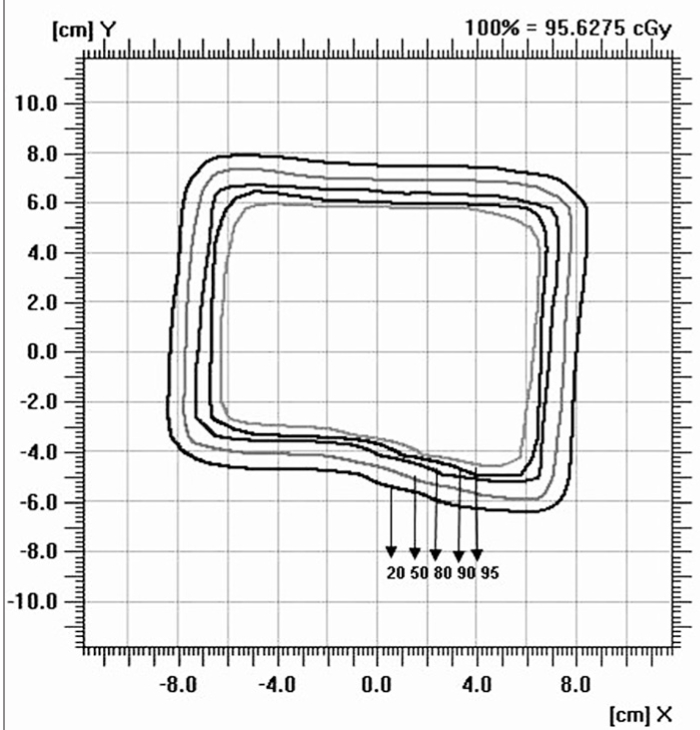
Isodose perpendicular to the beam central plane for 6-MeV electron beam at 100 cm SSD for 6.5 × 9 cm^2^ field size

**Figure 4b:**
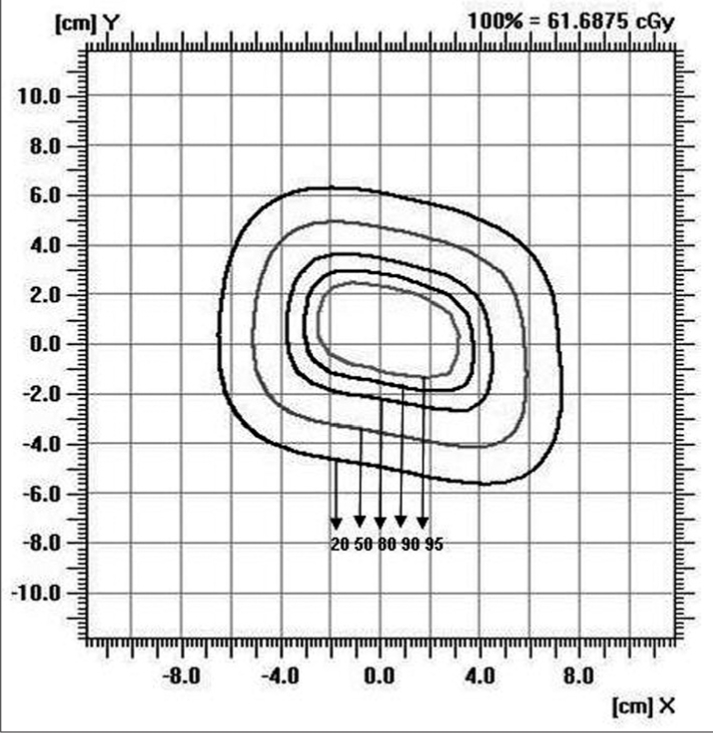
Isodose perpendicular to the beam central plane for 6-MeV electron beam at 120 cm SSD for 6.5 × 9 cm^2^ field size

**Figure 4c:**
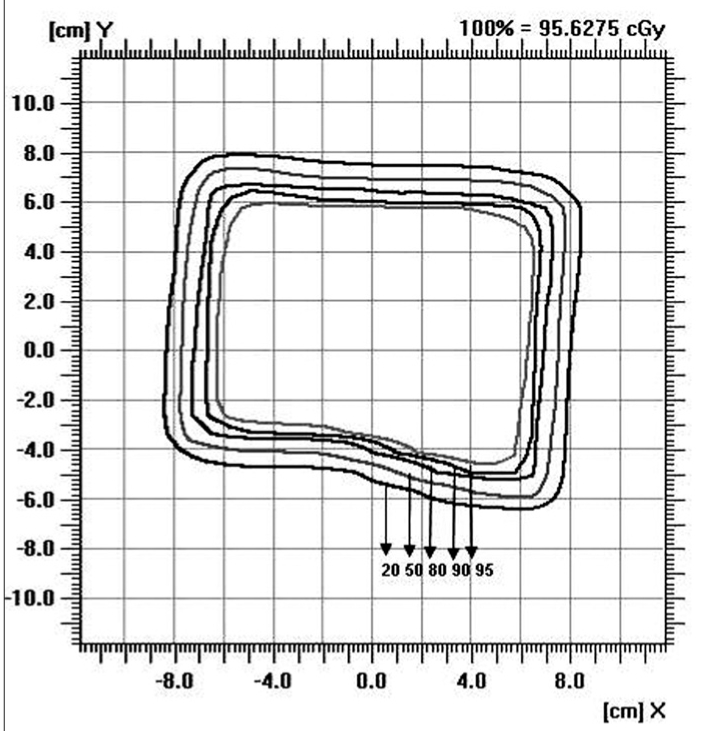
Isodose perpendicular to the beam central plane for 6-MeV electron beam at 100 cm SSD for 11.5 × 15 cm^2^ field size

**Figure 4d:**
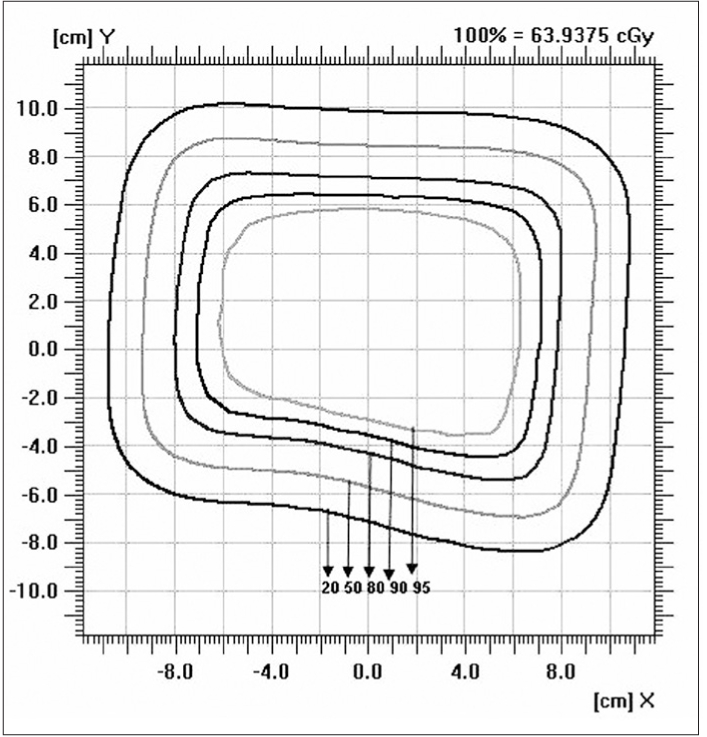
Isodose perpendicular to the beam central plane for 6-MeV electron beam at 120 cm SSD for 11.5 × 15 cm^2^ field size

### Uniformity index

Tables 3a and b shows the uniformity index values for the field sizes mentioned above and for various SSDs. The uniformity index should be > 0.7 for a field size greater than 10 × 10 cm^2^ as per ICRU-35.[6] The uniformity index deviation between 10 × 10 cm^2^ and 6.5 × 9.0 cm^2^ and for 6, 9, 12, 16, and 20 MeV at extended SSDs is shown in Table 3a. Similarly, the uniformity index deviation between 15 × 15 cm^2^ and 11.5 × 15 cm^2^ are shown in Table 3b. This shows that for smaller field sizes the beam was less uniform, and as the energy increased the uniformity also increased. The uniformity and flatness of the beam decreased for lower energies and for smaller fields because of the multiple scattering of the electron beam. For higher energies, where the scattering power of the electron beam is lower and the beam is more forwardly directed, the beam spread is very small. The decrease in uniformity indexwith increase in SSD was because of the large angular scattering of the electron beam, so that the clinically useful isodose level (80% and 90%) width decreases with SSD. This suggests that target coverage at extended SSD with irregular cut-outs may be inadequate unless relatively larger fields are used. For irregular cut-outs the beam characteristics has to be analyzed in order to deliver uniform dose to the tumor.

**Table 3a:** Uniformity index 10 × 10 cm^2^ and 6.5 × 9.0 cm^2^ field sizes for various energies

*SSD*	*6 MeV*		*9 MeV*		*12 MeV*		*16 MeV*		*20 MeV*	
	*10×10 cm^2^*	*6.5×9 cm^2^*	*10×10 cm^2^*	*6.5×9 cm^2^*	*10×10 cm^2^*	*6.5×9 cm^2^*	*10×10 cm^2^*	*6.5×9 cm^2^*	*10×10 cm^2^*	*6.5×9 cm^2^*
100	0.66	0.61	0.69	0.63	0.68	0.62	0.69	0.63	0.67	0.63
102	0.65	0.56	0.69	0.61	0.67	0.61	0.68	0.62	0.68	0.62
104	0.64	0.54	0.68	0.58	0.67	0.60	0.68	0.61	0.68	0.61
106	0.62	0.50	0.66	0.56	0.67	0.59	0.68	0.61	0.68	0.61
108	0.59	0.46	0.65	0.54	0.66	0.58	0.68	0.61	0.68	0.61
110	0.56	0.43	0.63	0.51	0.64	0.57	0.68	0.59	0.68	0.59
115	0.49	0.38	0.59	0.47	0.63	0.55	0.68	0.58	0.68	0.58
120	0.44	0.32	0.54	0.44	0.60	0.49	0.65	0.57	0.66	0.57

**Table 3b:** Uniformity index for 15 × 15cm^2^ and 11.5 × 15 cm^2^ field sizes for various energies

*SSD*	*6 MeV*		*9 MeV*		*12 MeV*		*16 MeV*		*20 MeV*	
	*15 × 15 cm^2^*	*11.5 × 15 cm^2^*	*15 × 15 cm^2^*	*11.5 × 15 cm^2^*	*15 × 15 cm^2^*	*11.5 × 15 cm^2^*	*15 × 15 cm^2^*	*11.5 × 15 cm^2^*	*15 × 15 cm^2^*	*11.5 × 15 cm^2^*
100	0.73	0.75	0.79	0.78	0.77	0.76	0.79	0.78	0.77	0.77
102	0.72	0.73	0.79	0.76	0.77	0.74	0.76	0.76	0.77	0.76
104	0.71	0.69	0.78	0.74	0.78	0.73	0.79	0.76	0.78	0.75
106	0.69	0.66	0.78	0.73	0.77	0.73	0.78	0.75	0.77	0.74
108	0.68	0.65	0.76	0.72	0.76	0.72	0.78	0.74	0.76	0.73
110	0.65	0.62	0.75	0.70	0.74	0.71	0.76	0.73	0.76	0.73
115	0.61	0.57	0.70	0.66	0.71	0.68	0.76	0.71	0.75	0.72
120	0.56	0.52	0.67	0.62	0.69	0.66	0.73	0.70	0.73	0.71

## Conclusion

Electron beam cutouts are used in the clinic to shape the beam used to treat small superficial lesions by conforming the shape of the radiation field to the tumor, while sparing dose to surrounding tissues and organs at risk. When the cut-out is used the field size becomes smaller, and that very small field may be inappropriate for treatment because of underdosage of lateral tissues. The result shows that while treating patients at extended SSD using electron cut-out the PDD, flatness, penumbra, and uniformity of the electron beam is affected. In particular, the higher isodose line constriction with respect to field size and SSD leads to underdosage of the treatment volume. In treatment planning and dose delivery, a slight offset of the field will also result in a large dose displacement with respect to the intended target. Most of the treatment planning systems are not suitable for extended SSD calculation. Finally, it may be worthwhile to implement the electron dosimetric data measured with cut-outs at different SSDs into the treatment planning system so that the oncologist and physicist have a clear idea regarding the appropriate energy and field size to be selected in order to deliver uniform dose to tumor volume.
